# Seismic analysis of Islamic Egyptian minarets through 3D scanning and dynamic simulation

**DOI:** 10.1038/s41598-024-69948-6

**Published:** 2024-09-02

**Authors:** A. M. Abdel-Wahab, Amr H. Badawy, M. S. El-Feky

**Affiliations:** Civil Engineering Department, Engineering & Renewable Energy Research Institute, National Research Centre, Cairo, Egypt

**Keywords:** Terrestrial laser scanner (TLS), Monuments documentation, Three dimensional dynamic analyses, Monuments stability, Earthquake behavior, Engineering, Materials science

## Abstract

Recently, Egypt had seismic activity. These seismic events have affected the stability of minarets, especially historical ones. Weight is one of the minaret's main stability factors. The main objective of the current research is to perform a three-dimensional (3D) assessment of an existing minaret, determine its accurate spatial model, document its current condition, examine its stability in the event of earthquakes, and identify the requisite measures to safeguard the minaret from any potential damage. The masonry to construct the minaret was used by extracting and examining specimens of this substance to determine its physical characteristics. The current work created three-dimensional models of the Abou-Ghanam El-Bialy minaret using a terrestrial laser scanner (TLS) to document its current condition, as well as minaret was subjected to a free vibration analysis using 3D finite element modeling. Finally, the minaret's seismic behavior was assessed utilizing mode forms, base responses, and normal stresses. The surveying method effectively documented the Minarets' existing case. The 3D seismic analysis showed that the minaret responded dynamically to earthquake loading, with mode shapes, base reactions, and normal stresses being crucial characteristics. Based on these data, we may suggest procedures to protect the minaret during seismic events.

## Introduction

Nowadays, Egypt is situated in seismic zones. These seismic events have a crucial impact on historical sites. The minaret represents the most extreme example of these constructions. It is well-known that Egypt possesses a significant number of minarets, particularly those of historical significance^1^. Each of the aforementioned minarets is ancient and built on a foundation of masonry. The stability of the minaret is derived from its mass. Furthermore, it has been observed that the majority of the current minarets are erect with a slight inclination. There are numerous methods available for doing three-dimensional surveys of monuments, as mentioned by^2–4^. The choice of a certain approach depends on various aspects, including the form and shape of the minaret, the permissibility of physical contact with the minaret, and the surrounding circumstances and limitations. Additionally, the measurement must be devoid of any significant and consistent mistakes. While it is not possible to eliminate random errors from measurements, they can be reduced by employing least squares adjustment processes^5^. A multitude of studies have been carried out to examine the effects of seismic and wind stresses on historical towers and minarets, shedding light on the structural vulnerabilities and resilience of these architectural marvels. Nohutcu (2019)^6^, Hoseynzadeh and Mortezaei (2021)^7^, and Dogangun et al. (2008)^8^ have contributed significantly by investigating the seismic impacts on brick masonry minarets, employing diverse ground motion scenarios to assess structural responses.

Moreover, Mortezaei et al. (2012)^9^, Romero et al. (2023)^10^, Ferraioli et al. (2017)^11^, and Ferraiolia et al. (2023)^12^, Işık et al. (2022)^13^, and Işık et al. (2023)^14^ have utilized advanced finite element analysis techniques to delve into the structural performance and seismic stresses affecting masonry minarets under static and dynamic conditions. Their studies have provided valuable insights into the behavior of such structures under varying loading scenarios, enhancing our understanding of their seismic resilience. Shakya et al. (2014)^15^, Shakya et al. (2018)^16^, and Acito et al. (2023)^17^ have adopted a simplified yet effective approach to evaluate the seismic efficacy of slender masonry minarets, offering practical insights into the seismic response of these architectural elements. Furthermore, Hejazi et al. (2016)^18^, Usta (2021)^19^, and Işık et al. (2022)^13,20^ have conducted detailed investigations on the structural response of nine brick masonry minarets, considering a range of factors such as weight, temperature variations, wind loads, and seismic events. Their use of nonlinear, three-dimensional finite element models has enabled a comprehensive analysis of the complex interactions influencing the stability and performance of these structures. In addition, Bayraktar et al. (2018)^21^ and Altıok and Demir (2021)^22^ have explored the impact of intense vertical ground motion on tall historical masonry minarets through a series of rigorous linear and nonlinear time history analyses. Their investigations into horizontal and combined horizontal-vertical ground motion scenarios have provided crucial insights into the dynamic behavior and vulnerabilities of such architectural elements under varying seismic conditions. The structural integrity of minarets is often challenged by various forms of damage, including cracks in masonry elements, displacement of structural components, weakening of mortar joints, and overall structural deformation. These damages not only compromise the aesthetic appeal of the minaret but also raise concerns about its structural stability and long-term preservation. Understanding the nature and extent of these damages is crucial in formulating effective restoration and conservation strategies to ensure the continued integrity and authenticity of these architectural treasures for future generations to admire and appreciate has been studied previously by Işık et al. (2023)^23^.

The primary goal of the present study article is to conduct three-dimensional surveys of the existing minaret in order to determine its precise spatial model for the purpose of recording its current state. Next, investigate the structural integrity of these minarets during seismic events, and identify the necessary measures to prevent any damage to the minarets.

## Field experiments and instrumentation

### Historical background

The Abu Ghannam Mosque, situated in the heart of the city of Bela in Kafr El-Sheikh Governorate, is one of the oldest and most renowned religious structures in the region. Constructed in 700 AH (approximately 1300 CE) during the Mamluk era, the mosque is widely regarded as an architectural masterpiece. The mosque's minaret, an integral component of the complex, was commissioned and built concurrently with the main structure under the patronage of the Mamluk Sultan Al-Nasir Muhammad ibn Qalawun. This towering architectural feature has stood as a prominent landmark for over seven centuries, serving as a testament to the skilled craftsmanship and ingenuity of its Mamluk-era builders. Throughout its long and storied history, the minaret has faced various challenges, including damage from natural disasters and the gradual effects of weathering and environmental factors. Moreover, the minaret has not undergone any significant restoration efforts in the past to address structural issues or preserve its architectural integrity. The mosque's founder, Sidi Salem Al-Bili Abu Ghannam, was a revered saint and spiritual leader whose legacy continues to be celebrated and revered by the local community. The Abu Ghannam Mosque, with its iconic minaret, remains an important center of religious and cultural significance in the Kafr El-Sheikh region, embodying the rich historical and architectural heritage of the Mamluk era.

### Layout and geometry of the minaret

**Figure 1:**
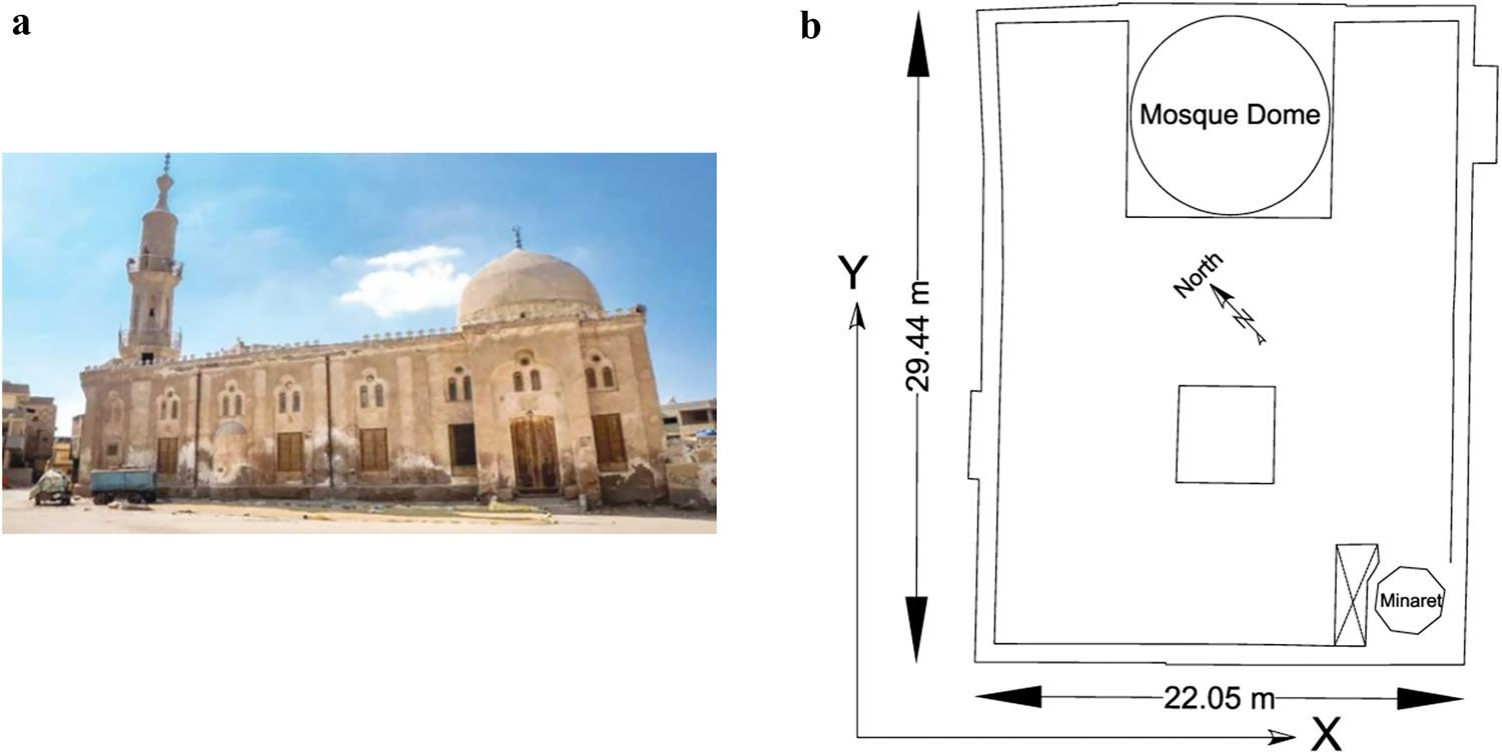
(**a**) Abou-Ghanam El-Beily Mosque. (**b**) Plan of Abou-Ghanam El-Beily Mosque.

The Abou-Ghanam El-Biely Minaret has been surveyed. The mosque was constructed in the year 700, as per the Hijri calendar, which corresponds to 744 years ago, and its precise position is at latitude 31° 10′ 32.75″ N and longitude 31° 12′ 58.036″ E, based on the WGS84 datum. This mosque is officially designated as a monument. Figure 1a and b depicts an image of the front facade of the mosque. This photograph clearly depicts the mosque, its foundation, and minaret, all constructed using masonry. Furthermore, the minaret of the mosque exhibits a prominent slant. The stability of the minaret is solely derived from its own weight in relation to its foundation.

### Instrumentation and field experiments setup

Prior to use, all instruments utilized in the experiment are calibrated and adjusted.

The Trimble TX8 Laser Scanner with Extended Range is available.

**Figure 2:**
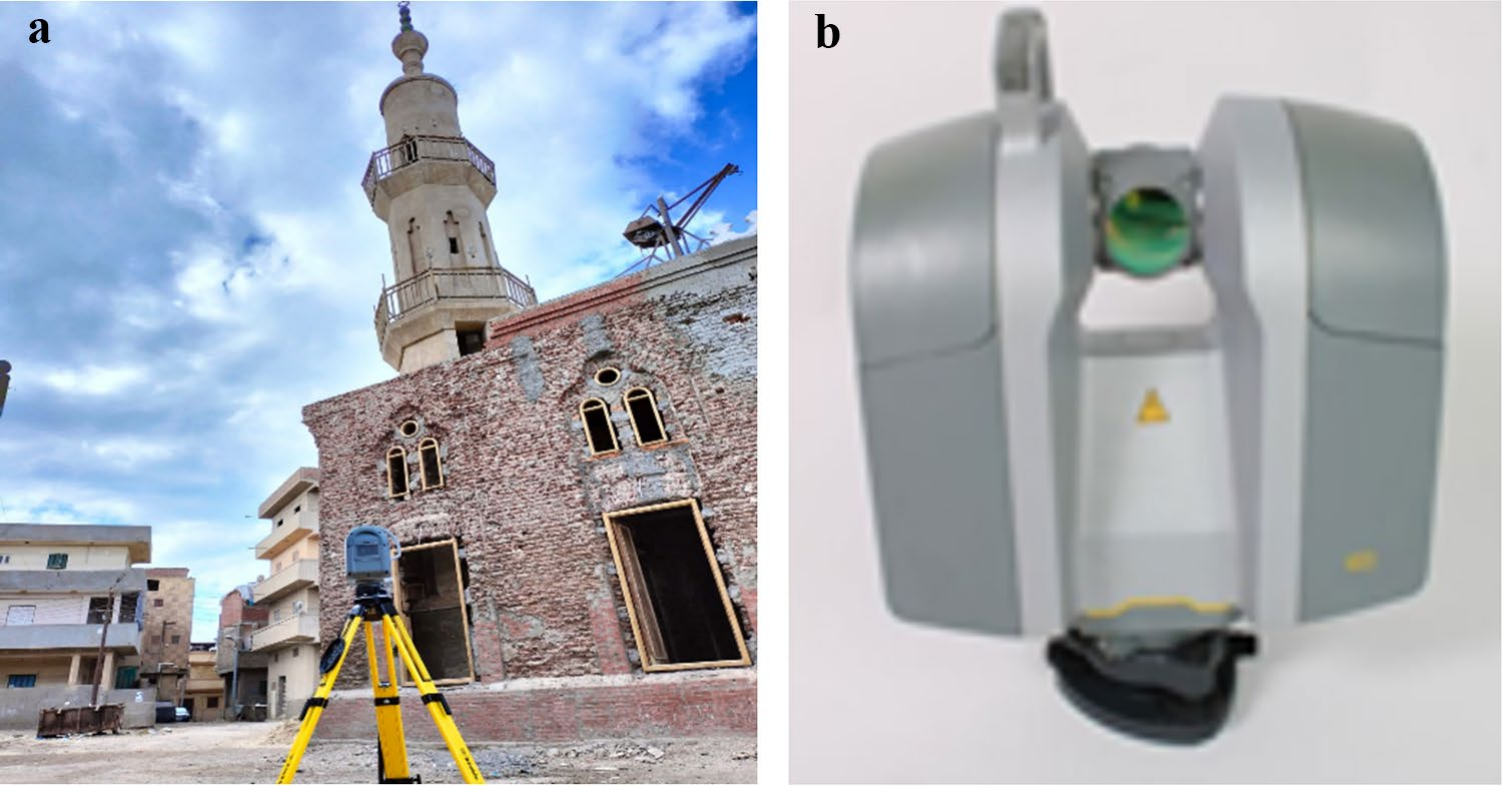
(**a**) Trimble TX8L Terrestrial Laser Scanner in sit. (**b**) Trimble TX8L Terrestrial Laser Scanner.

This scanner is a fast and efficient 3D laser scanner^24^. The device is capable of measuring speeds up to 1,000,000 points per second and has a range of 340 m. Additionally, there is a built-in colour camera, as depicted in Fig. 2a and b.

2-Control Point

**Figure 3:**
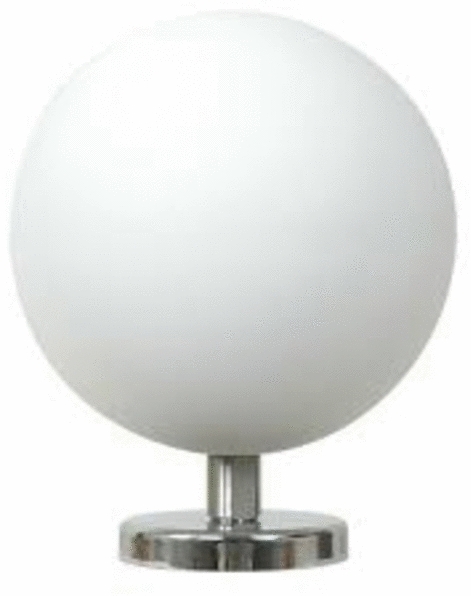
Control point shape.

The control point is represented by a magnetic sphere. Figure 3 illustrates the shape of the control point.

A total of ten control points, shaped like spheres, were securely positioned around the minaret. The minaret was surveyed from 7 different places, ensuring that each scan had a minimum of 4 control points.

### Data processing and 3D model

**Figure 4:**
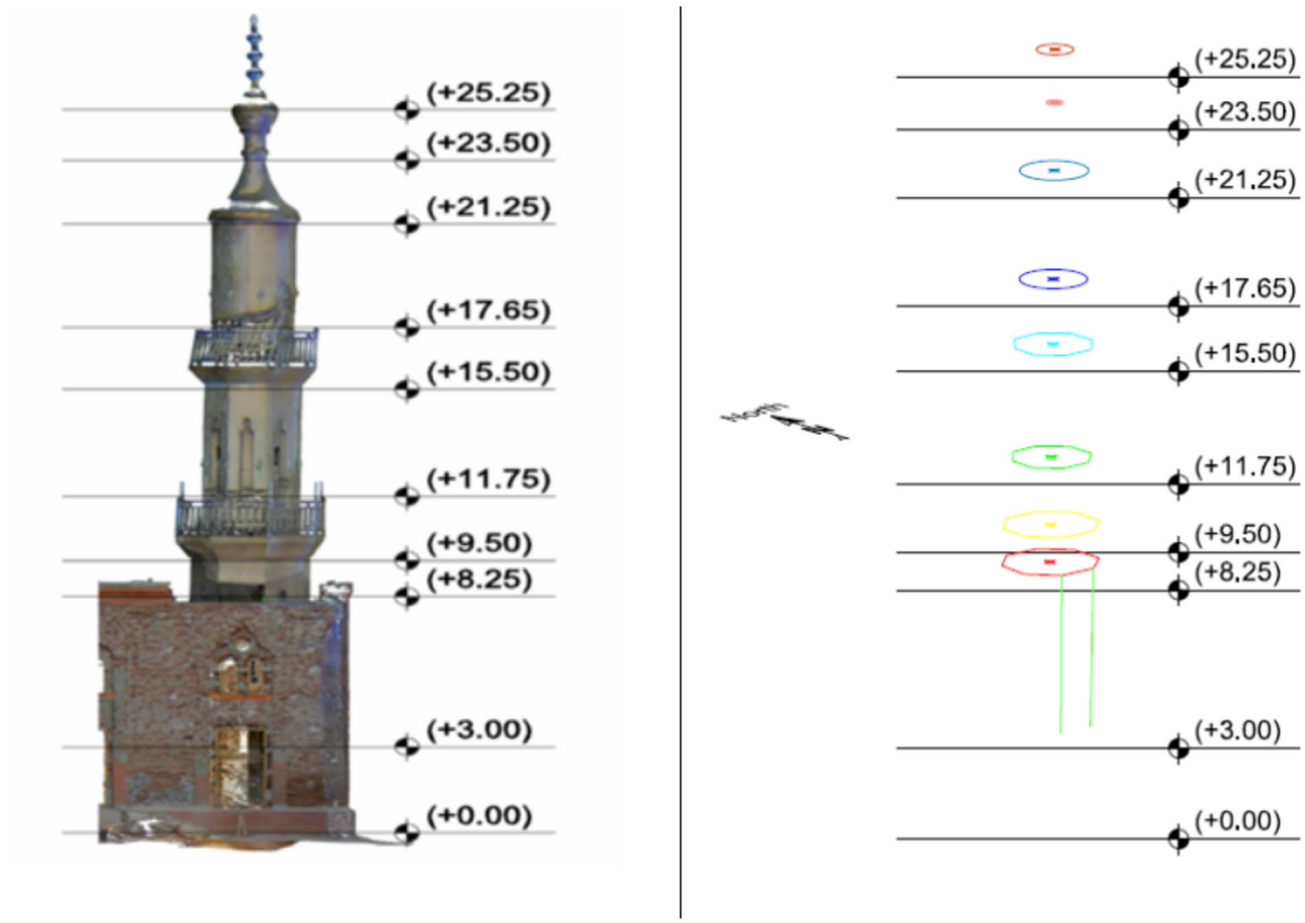
Shape of the obtained 3-D model of the minaret and the position of the nine sections.

The Trimble Real World Programme environment automatically registered and geo-referenced the obtained data to construct a three-dimensional model of the minaret. The next step involves transferring the model to the Autodesk Recap environment^25,26^. Nine distinct sections comprise the three-dimensional model of the minaret, as seen in Fig. 4.

**Figure 5:**
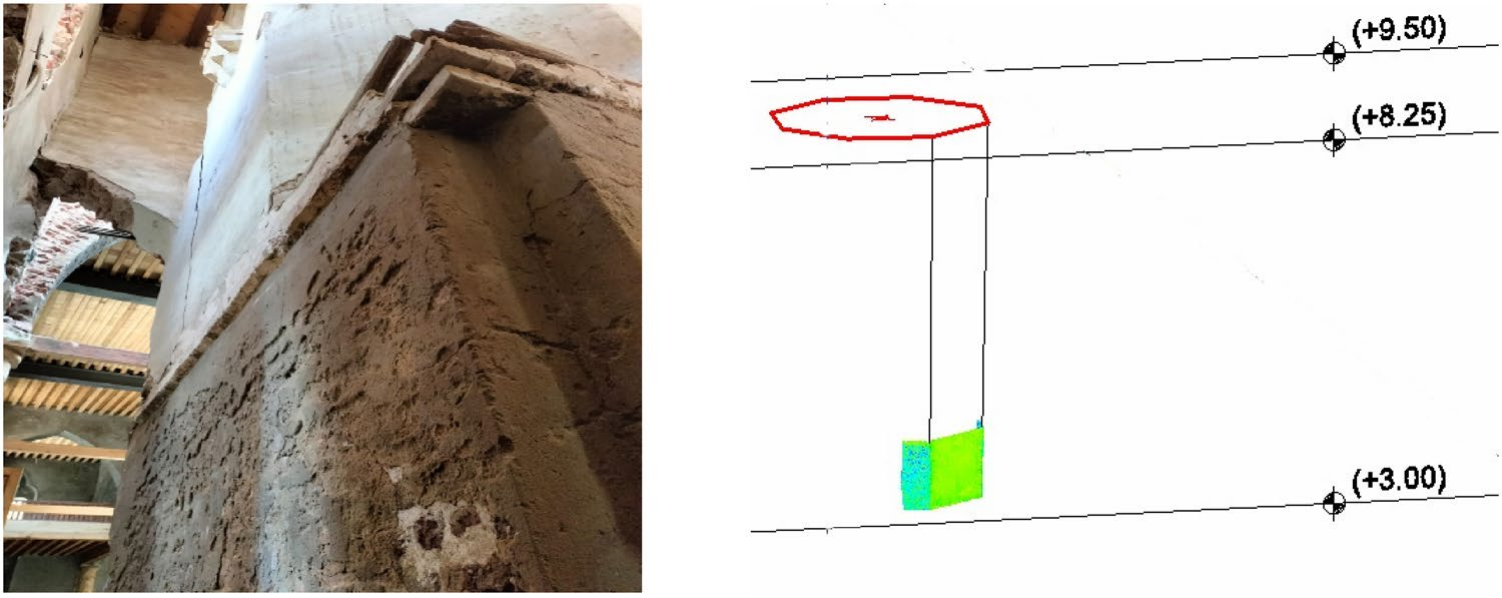
The two edges appeared at the lower part of the minaret.

The centre of gravity of each section is calculated. Following this, the inclination of the minarets is calculated for both the bottom and upper sections of the minarets. The bottom half of the minaret was analyzed to determine the inclination of the edges at section 1 and section 2, as shown in Fig. 5 and Table 1.

**Table 1:** The inclination between section (1) and section (2).

ΔX (cm)	ΔY (cm)	ΔP (cm)	ΔZ (m)
5.4	−7.9	9.6	5.25

For the upper part, the position of the center of gravity is computed for each section. Then, the inclination is computed as appeared in Table 2.

**Table 2:** The coordinates of the center of gravity and the inclination of the center of the minaret.

Section no.	Center coordinates			Differences in coordinates and position			
	X (m)	Y (m)	Z (m)	ΔX (cm)	ΔY (cm)	ΔP (cm)	ΔZ (m)
2	330.585	199.328	8.25				
3	330.602	199.311	9.50	1.7	−1.7	2.4	1.25
4	330.626	199.264	11.75	4.1	−6.4	7.6	3.50
5	330.686	199.233	15.50	10.1	−9.5	13.9	7.25
6	330.694	199.257	17.65	10.9	−7.1	13.0	9.40
7	330.719	199.252	21.25	13.4	−7.6	15.4	13.00
8	330.738	199.265	23.50	15.3	−6.3	16.5	15.25
9	330.743	199.285	25.25	15.8	−4.3	16.4	17.00

**Figure 6:**
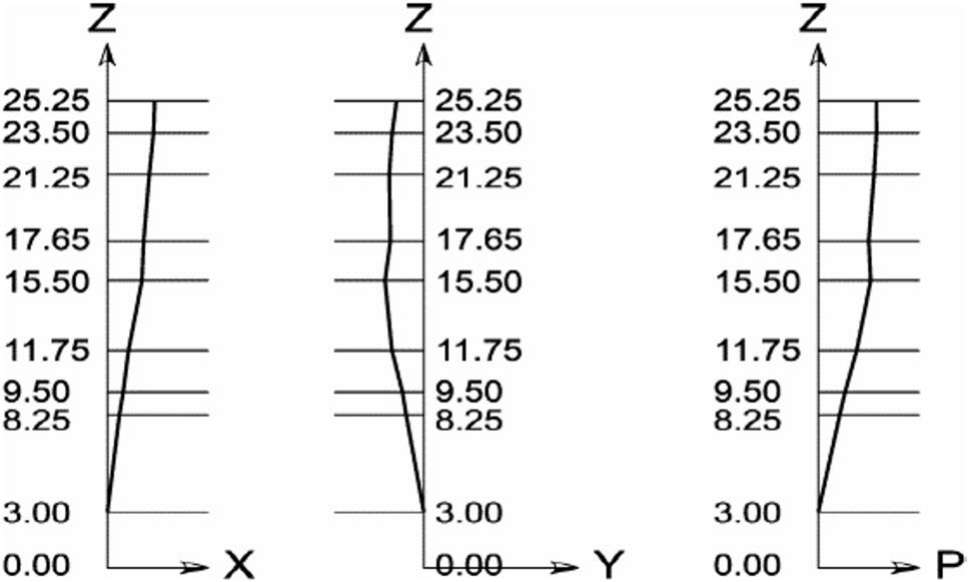
The inclination of the center of gravity of the minaret.

From the results of Tables 1 and 2, the inclination of the minaret is computed and outlined in Fig. 6.

As a closing remark, the minaret is inclined 26 cm in vertical distance of 22.25 m, with an azimuth of the inclination equals 149°.

## Structural analysis and 3D modelling

### Modeling and analysis of minaret using finite element method

**Figure 7:**
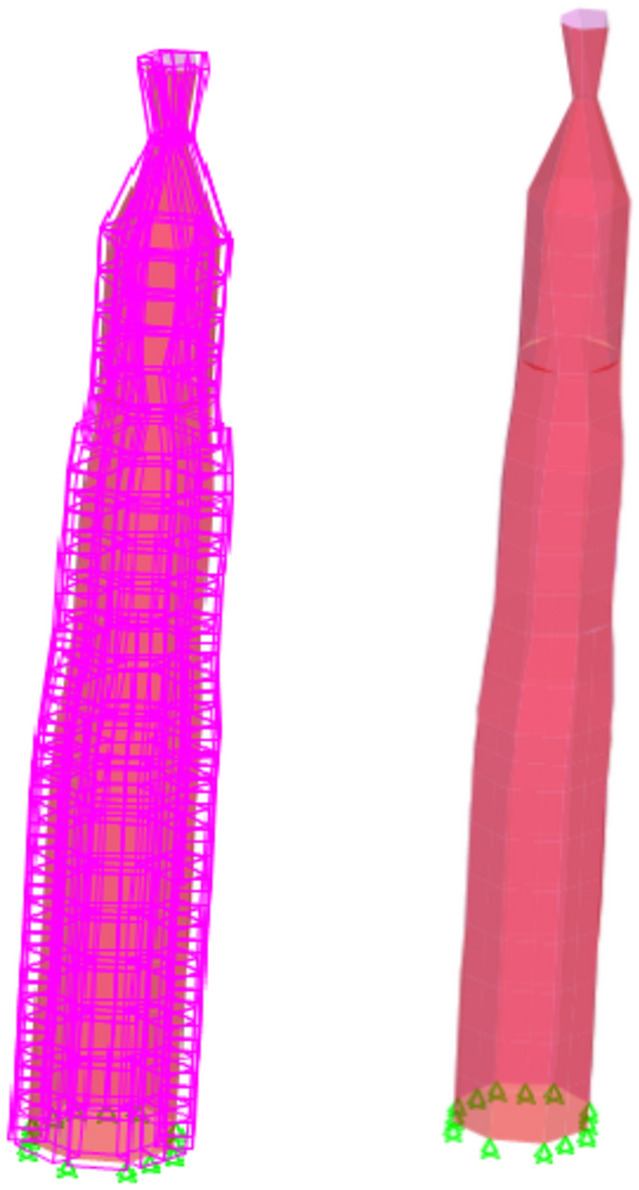
Sap2000 3D representation of the minarets up to section + 25.25.

The minarets were designed utilizing the shell element feature of the SAP2000 finite element software^27^. The purpose of this four-node element is to accurately represent and simulate three-dimensional structures (CSI, 2008). The proportions of the shell element were meticulously chosen to provide an aspect ratio close to unity in all three directions. The model incorporated the minarets' inclination, using the survey data. In order to replicate the soil condition, the movements in all three dimensions were constrained at the base of the minarets. This was accomplished by securing the joints at the foundation. The software computed the self-weight of the minarets as well as the masses of the joints. Figure 7 depicts a three-dimensional portrayal of the minarets up to section + 25.25. The minaret model consisted of 3800 elements.

### Minaret's brick properties

**Figure 8:**
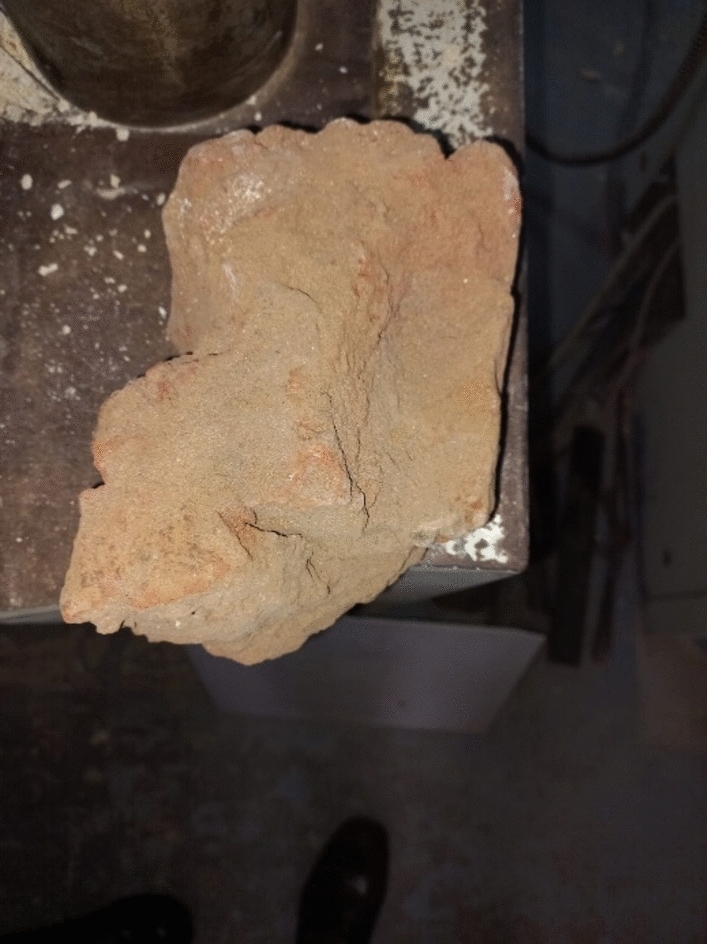
Minaret's red masonry brick sample.

**Figure 9:**
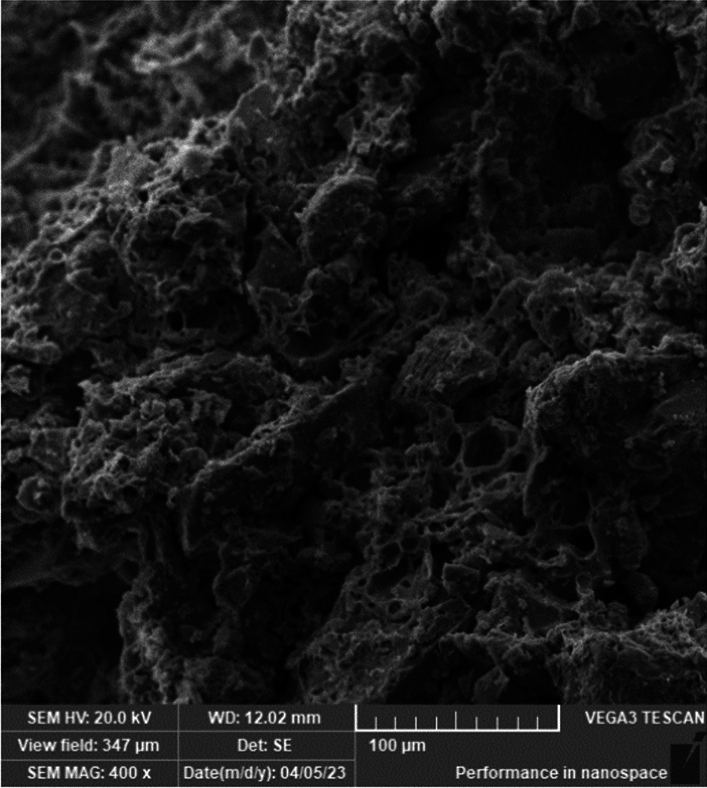
SEM brick micrograph.

The minarets were built utilizing Red Masonry bricks, as depicted in Fig. 8. In order to ascertain the physical characteristics of these bricks, real samples were collected and meticulously examined and evaluated at the Laboratory located at the National Research Centre in Egypt. Figure 9 displays the unprocessed block that was captured before undergoing the sharpening and testing procedures.

The samples were subjected to compression testing following the European Standard EN 1926:2006 in order to ascertain their compressive strength. The flexural strength was assessed using the European Standard EN 12372:2006. The density of the granite was determined in accordance with the European Standard EN 1936:2006. The test findings were used to feed data into the model in order to determine the material properties of the minarets. Table 3 displays the specific characteristics of the minarets utilized in the model.

**Table 3:** Material properties of the minaret's Bricks.

Material used	Red masonry brick
Density (t/m^3^)	1.4
Modulus of elasticity (t/m^2^)	696,906
Compressive strength (MPa)	22
Tensile strength (MPa)	3.8
Poisson’s ratio	0.29
Coefficient of friction	0.27

As for the mechanical properties of the brick material, the study incorporated these properties as inputs in the model's material properties section. Furthermore, stress outputs were meticulously compared against the brick's strength properties to ensure the minaret's stiffness and structural integrity. This meticulous verification process confirms that the model effectively accounts for the brick material's behavior and its contribution to the overall structural performance of the minaret.

**Figure 10:**
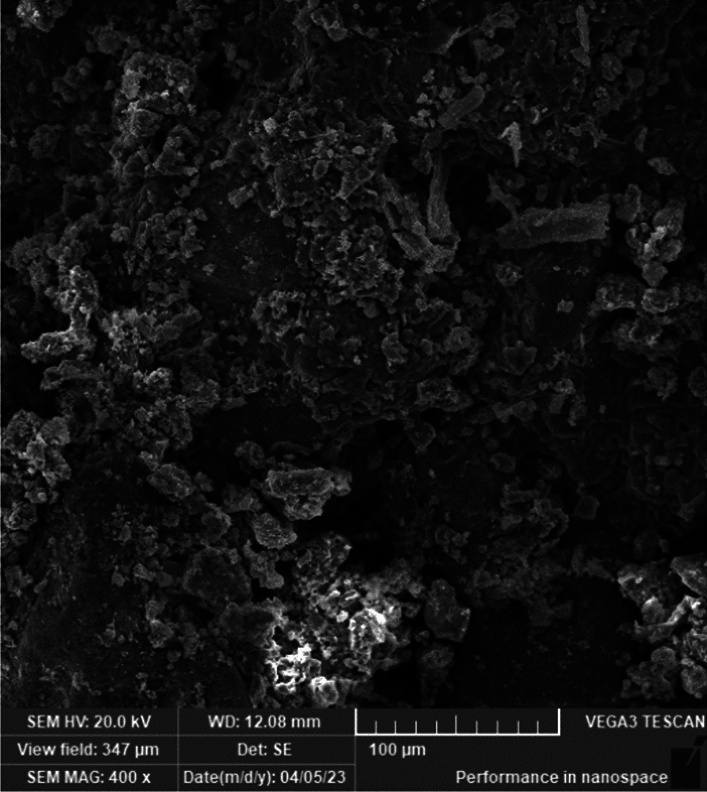
SEM mortar micrograph.

The scanning electron microscope micrographs in Fig. 9, and Fig. 10 found using TESCAN – VEGA3 Scanning electron microscopy^28^ depict the actual samples of bricks and mortars. These figures demonstrate the amorphous porous structure of the bricks, which allows for effective filling with the mortar. This results in a highly interlocking performance between the bricks and mortar. Additionally, the micrograph of the mortar reveals a more compacted matrix with a curved zigzagged surface, indicating interlocking within the pores of the brick surface.

SEM images in Figs. 9 and 10, these images serve a crucial purpose in elucidating the microstructural characteristics of the materials used in the Finite Element Model. The SEM images offer valuable insights into the microscopic morphology, composition, and quality of the materials, contributing to a better understanding of their behavior. In particular, these images assist in visualizing possible microstructural defects, interfaces, and bonding mechanisms within both the brick and mortar materials. This visualization is crucial for accurately capturing the complete bond between them, given their amorphous surfaces, which is essential for assuming a unified material response in the FEM analysis. By including these SEM images, we enhance the comprehensiveness and validity of the model, ensuring a more detailed and informed analysis of the minaret's structural performance.

### Response surface

**Figure 11:**
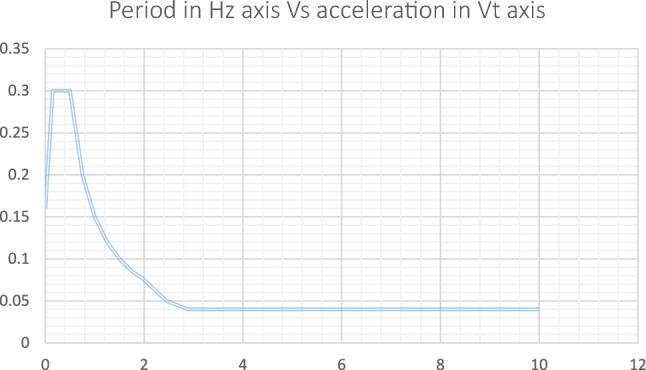
Sap 2000 shows the input of the response surface applied.

The reaction spectrum is a useful instrument in scientific study for analyzing and evaluating how structures respond to seismic loads. Researchers can acquire a full understanding of the structural behaviour under seismic excitation by graphically displaying the maximum response of a structure at different frequencies, considering the features of the underlying ground motion. By graphing the highest recorded reactions in terms of acceleration, velocity, or displacement against the associated frequencies, it is possible to identify important vibration patterns and evaluate the structural performance and safety. This approach is very beneficial in the seismic design and evaluation of structures, as it allows researchers to optimize structural designs, choose suitable materials, and implement effective mitigation techniques. The aim of this research study is to examine how the response spectrum affects the location of minarets. Specifically, we will analyze the maximum response of the minarets at various frequencies, while considering the characteristics of the seismic ground motion. The results of the response spectrum analysis will offer significant information about the dynamic behaviour of the minarets during an earthquake. This analysis will help identify the most important modes of vibration and their related maximum responses. Furthermore, it will simplify the assessment of the structural integrity and seismic resilience of the minarets. Figure 11 depicts the input of the Response surface, which is utilized based on the seismic circumstances at the location of the minaret.

### Finite element analysis

To analyze the effects of the earthquake on the minaret, several factors were investigated, such as mode forms, base reactions, and axial normal stresses.

#### Mode shapes

**Figure 12:**
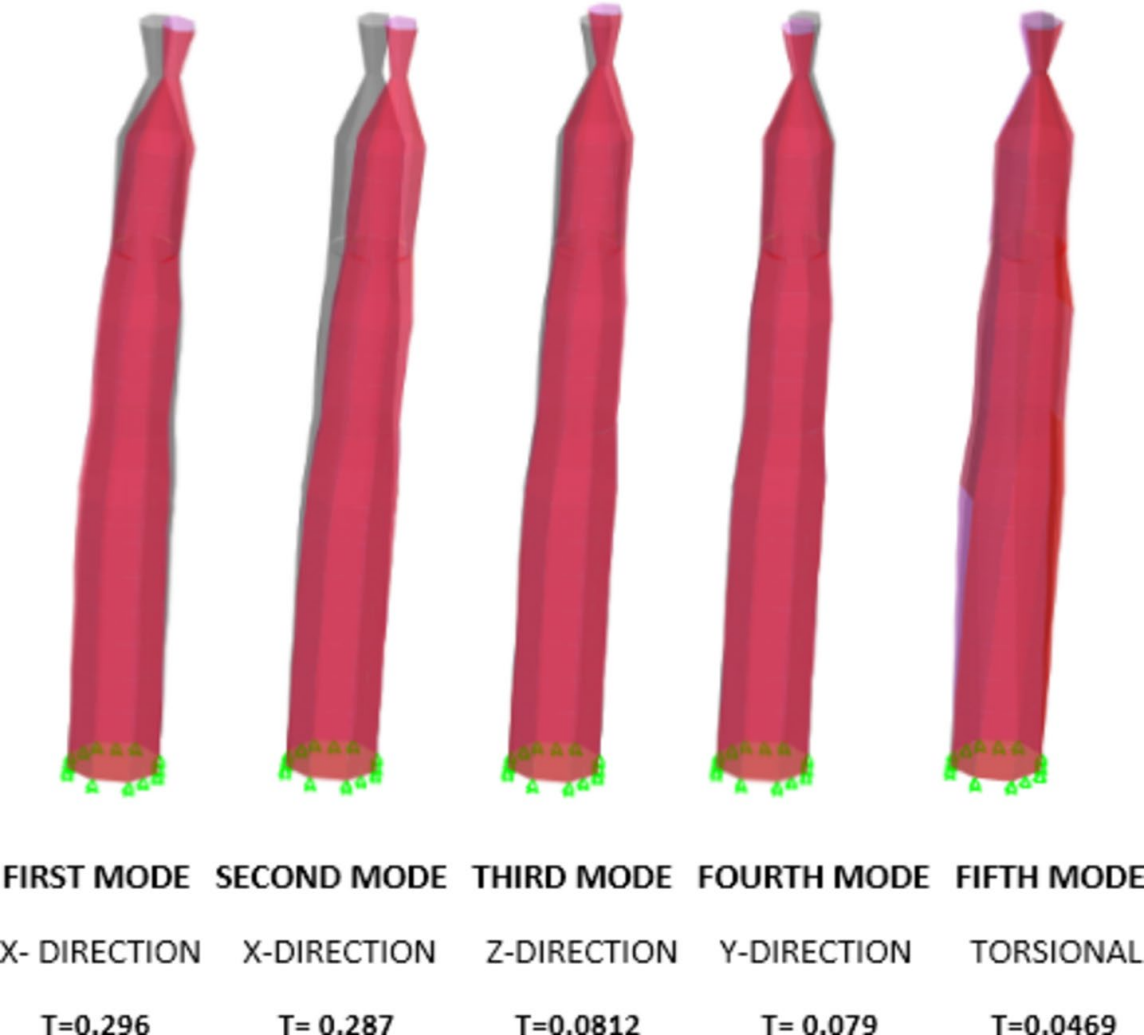
Presents the first four bending modes & the fifth torsional mode.

In order to examine the characteristics of the minaret, a 3D finite element modelling method was used to subject it to free vibration analysis. The analysis specifically targeted the initial five modes of vibration. Significantly, the mode shapes displayed a constant sequence across the minaret, suggesting a commonality in their dynamic response. Figure 12 visually illustrates the first four bending modes and the fifth torsional mode, offering a full comprehension of the unique vibration patterns displayed by the minaret. The mode shapes followed an almost similar pattern as mentioned by^29^. In order to verify the model the following empirical equations were considered^30,31^

1 $$ {\text{T1}} = 0.0187*{\text{H}} $$

2 $$ {\text{T1}} = 0.01137*{\text{H}}^{1.138} $$

The values from the above equations are as follows:

- T1 from model = 0.296
- T1 from Eq. (1) = 0.374
- T1 from Eq. (2) = 0.342

In the validation process of the model, two empirical equations were employed for comparison. Equation 1 (T1 = 0.0187H) and Eq. (2) (T1 = 0.01137H^1.138^) were considered for this analysis. The calculated values resulting from these equations are as follows: the model-generated T1 value stands at 0.296, while Eq. (1) yields a T1 value of 0.374 and Eq. (2) provides a T1 value of 0.342. This comparison reveals a slight disparity between the model-predicted value and the values obtained from the empirical equations. The outcomes suggest that while the model's estimation closely aligns with the value from Eq. (2), there exists a notable deviation when compared to the result derived from Eq. (1).

#### Results of the stresses affecting the minaret's structure

A comprehensive three-dimensional structural analysis was meticulously conducted on the minaret, taking into account its current inclination and incorporating the specified resistance parameters outlined in the report as well as those provided by the executing company. Notably, the upper segment of the minaret, deemed non-critical to its structural integrity, was intentionally excluded from the analysis to focus solely on the essential load-bearing components. Subsequently, the structural system underwent a rigorous evaluation under seismic loading conditions to assess its robustness and resilience in the face of dynamic forces. The outcomes of this comprehensive analysis are summarized as follows:

### Finite element analysis results

**Figure 13:**
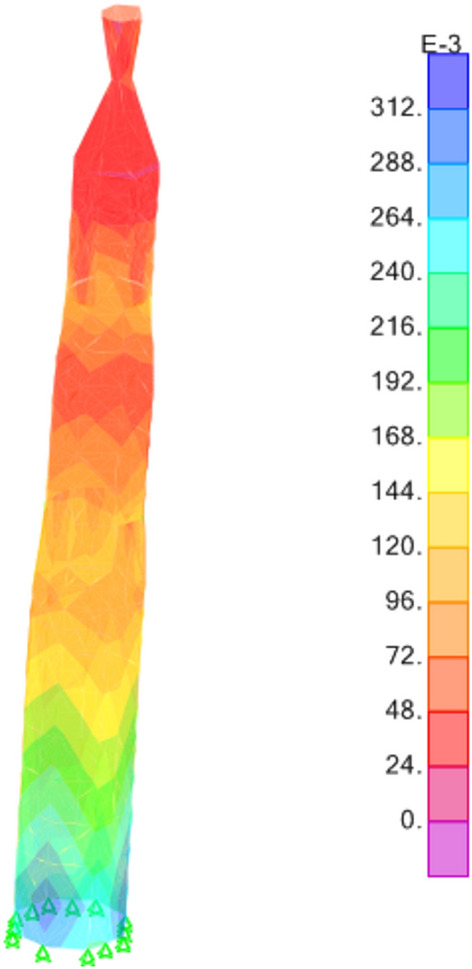
S11 Results (Mpa).

**Figure 14:**
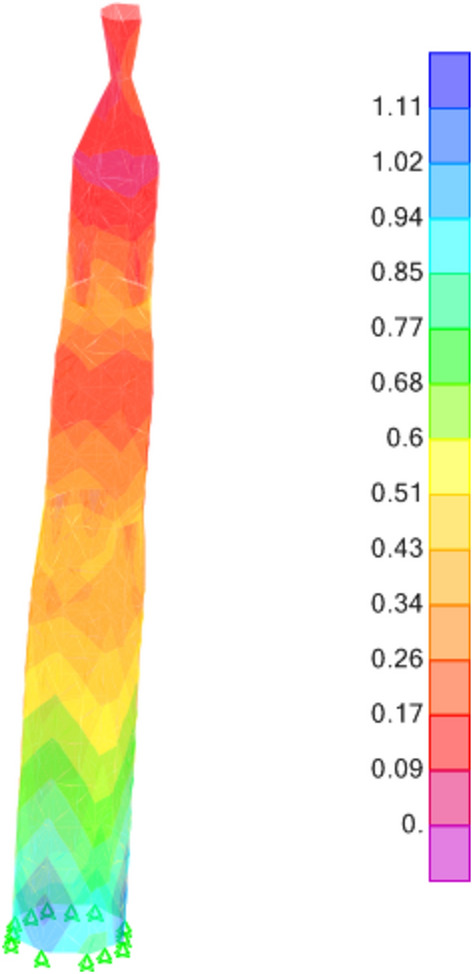
S22 Results (Mpa).

**Figure 15:**
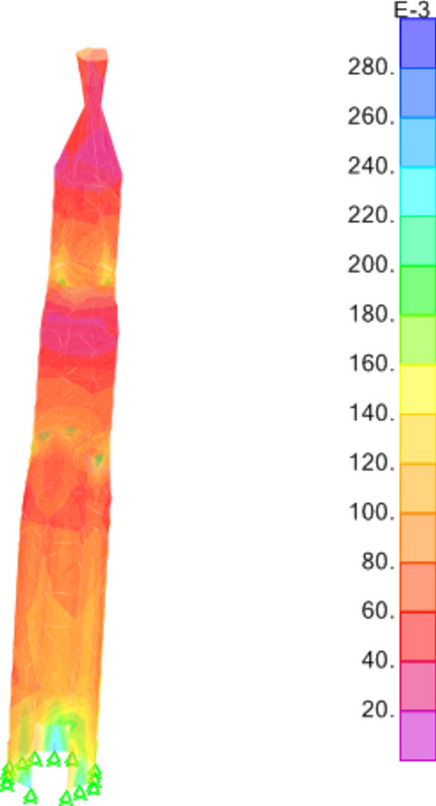
S12 Results (Mpa).

The results derived from the finite element analysis unveil critical insights into the distribution of stresses within the minaret's structure. Figures 13, 14, and 15 intricately illustrate the tensile stresses (S11 and S22) and shear stresses (S12) induced by G+EQx and G+EQy loadings. These stress distributions not only elucidate the internal forces acting within the structure but also reveal that the base shear force exerted on the minaret amounts to 39% of the total weight in both the x and y directions. This observation underscores the significance of load distribution and structural response under seismic conditions.

Furthermore, a closer examination of the stress outputs reveals that the maximum stresses experienced by the minaret are well within the material strength properties of the red masonry bricks. The stresses in the minaret have a factor of safety of almost 3.8 compared to the brick's strength values. This indicates a significant margin of safety in the structural design. However, it is important to note that the authors have also considered the findings from a previous study^32^, that in idealization of the structural model, the ideal brickwork should be assumed, since brick masonry is a layered material. For design purposes, the compressive strength of brick masonry obtained from finite element analysis should be increased by a value of 1.4, in order to obtain the actual strength of the brickwork. When this factor is applied, the factor of safety against the brick's strength properties is reduced to 2.7, which is still considered a sufficient safety margin for the minaret's structural integrity.

### Anticipated displacement analysis

**Figure 16:**
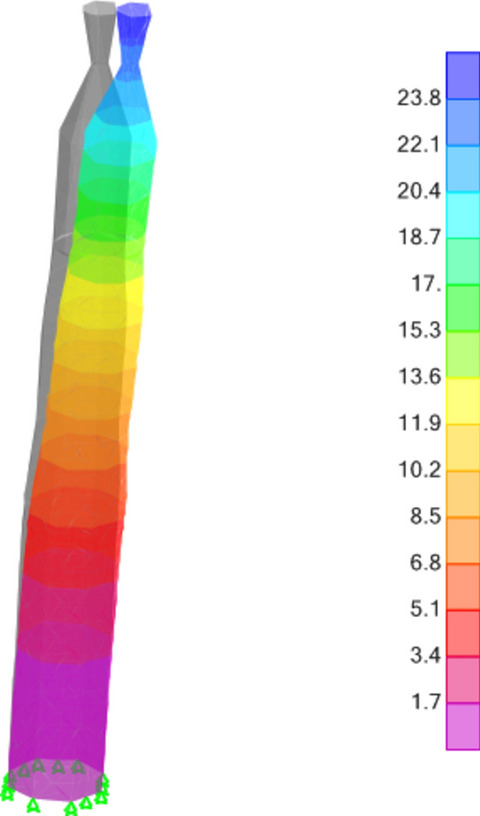
The maximum displacements are recorded at G + EQ (mm).

The anticipated increase in displacement of the minaret during seismic events was thoroughly examined to gauge its structural performance under dynamic loading. The maximum additional displacements were meticulously quantified as Δx=16 mm and Δy=16 mm along the x and y axes, respectively, as visually depicted in Figure 16. These displacement values serve as crucial indicators of the structure's flexibility and response to seismic excitations, providing essential information for understanding its behavior under varying loading conditions.

Finally the base shear reactions of the total combination is presented in the following Table 4.

**Table 4:** Base reactions.

Output case	Case type	Step type	Global FX	Global FY	Global FZ	Global MX	Global MY	Global MZ
Text	Text	Text	Tonf	Tonf	Tonf	Tonf-mm	Tonf-mm	Tonf-mm
COMB1	Combination	Max	34.014	34.2157	155.0791	31063145.97	−49773095	13242689.07
COMB1	Combination	Min	−34.014	−34.2157	150.9417	29929064.67	−51410313	−13242643.6

## Discussion and conclusion

### Structural observations and fractures

From the information provided above, the following conclusions can be drawn:

The use of high-resolution 3D laser scanners is very advantageous in generating 3D models, particularly in the case of historical structures that pose challenges for standard surveying methods.

The investigation highlights a notable association between minor fractures within the minaret and its tilting motion. This correlation underscores the significance of scrutinizing the morphology and alignment of fractures concerning the underlying displacements of the foundation. Such observations provide valuable insights into the structural behavior and potential vulnerabilities of the minaret.

### Assessment of upper sections and brick resistance

Noteworthy findings reveal that the upper sections of the minaret exhibit structural integrity devoid of visible flaws. Concurrently, the analysis indicates a relative weakness in the resistance of the bricks utilized, a crucial aspect discerned from the results obtained through sample testing. These insights shed light on the material performance and durability aspects critical to the minaret's stability.

### Structural analysis and seismic performance

The structural analysis conducted elucidates that the forces acting upon the minaret align within acceptable thresholds, signifying a level of structural robustness. Furthermore, projections suggest a potential 10% to 20% increase in relative displacement under seismic loading conditions, considering the foundational stability. These assessments lay the groundwork for understanding the structure's response to dynamic forces and its capacity to withstand seismic events.

### Seismic resilience evaluation

The primary aim of evaluating the seismic resilience of the Islamic Egyptian minaret was effectively addressed through a comprehensive finite element analysis tailored for seismic scenarios. Detailed assessments reveal that while maximum deformations hint at limited flexibility, peak stress values affirm the structural integrity and security of the minaret. Mode periods further validate the structural stiffness, while adherence to compression and shear stress limits prescribed by the Egyptian Earthquake Code underscores the structure's resilience.

### Material attributes and structural behavior

The utilization of material attributes derived from authentic masonry samples emerges as a pivotal aspect of the investigation. This crucial consideration accentuates the stability of the structure, unaffected by potential material degradation or a decline in component quality resulting from the brick–mortar connections. The maintained stress and displacement values within acceptable ranges, even under diverse considerations, collectively suggest the absence of significant structural concerns.

### Structural strength under seismic forces

In alignment with the analytical principles employed, the study affirms that the structure exhibits resilience against potential earthquake forces, showcasing a robust capacity to withstand such dynamic loads without substantial risk of damage or failure. This assessment underscores the inherent strength and stability of the minaret in the face of seismic challenges.

## Data Availability

All data generated or analysed during this study are included in this published article.
